# Physiological aspects of Toll‐like receptor 4 activation in sepsis‐induced acute kidney injury

**DOI:** 10.1111/apha.12798

**Published:** 2016-10-08

**Authors:** S. B. Anderberg, T. Luther, R. Frithiof

**Affiliations:** ^1^ Department of Surgical SciencesSection of Anesthesia & Intensive CareUppsala UniversityUppsalaSweden

**Keywords:** acute kidney injury, AKI, renal, sepsis, TLR4, Toll‐like receptor

## Abstract

Sepsis‐induced acute kidney injury (SI‐AKI) is common and associated with high mortality. Survivors are at increased risk of chronic kidney disease. The precise mechanism underlying SI‐AKI is unknown, and no curative treatment exists. Toll‐like receptor 4 (TLR4) activates the innate immune system in response to exogenous microbial products. The result is an inflammatory reaction aimed at clearing a potential infection. However, the consequence may also be organ dysfunction as the immune response can cause collateral damage to host tissue. The purpose of this review is to describe the basis for how ligand binding to TLR4 has the potential to cause renal dysfunction and the mechanisms by which this may take place in gram‐negative sepsis. In addition, we highlight areas for future research that can further our knowledge of the pathogenesis of SI‐AKI in relation to TLR4 activation. TLR4 is expressed in the kidney. Activation of TLR4 causes cytokine and chemokine release as well as renal leucocyte infiltration. It also results in endothelial and tubular dysfunction in addition to altered renal metabolism and circulation. From a physiological standpoint, inhibiting TLR4 in large animal experimental SI‐AKI significantly improves renal function. Thus, current evidence indicates that TLR4 has the ability to mediate SI‐AKI by a number of mechanisms. The strong experimental evidence supporting a role of TLR4 in the pathogenesis of SI‐AKI in combination with the availability of pharmacological tools to target TLR4 warrants future human studies.

Toll‐like receptors (TLRs) are crucial for sensing invading microorganisms like bacteria, virus and fungi. As a first line of defence, they initiate an innate immune response that includes activation of polymorphonuclear leucocytes (PMN), monocytes and macrophages (Medzhitov 2001, Hayashi *et al*. 2003, Farina *et al*. 2004). They also mediate the release of pro‐inflammatory cytokines and interferons with the ultimate goal to identify and destroy the pathogen (Medzhitov 2001). However, activation of the innate immune system sometimes also causes collateral damage to host tissue and cells, resulting in organ dysfunction and death. In this review, we explore how one specific TLR, Toll‐like receptor 4 (TLR4), may cause acute kidney injury (AKI) in sepsis. Although many TLRs have been described, TLR4 is so far the most extensively studied. A variety of experimental studies have shown beneficial survival effects of blocking this receptor in sepsis. But, as will be briefly outlined in this review, the data from the clinical setting are thus far not convincing.

Toll‐like receptors have previously been implicated as important mediators of disease in various forms of chronic and acute renal failure (Gluba *et al*. 2010, Valles *et al*. 2014). Here we will review the experimental evidence supporting a role for TLR4 in sepsis‐induced AKI (SI‐AKI) with emphasis on described mechanisms of action. As will become evident, TLR4 activation may affect both glomerular and tubular processes and experimental data describes a significant improvement in renal function if TLR4 is inhibited in sepsis.

## The Toll‐like receptor family

Ten different types of TLRs have been described in humans (TLR1‐10) (Matsushima *et al*. 2007). They are listed in Table 1 together with known ligands and their presumed role in SI‐AKI. Although TLRs are expressed in most human tissue, they predominate in organs and tissues typically involved in the immune defence such as spleen and blood, as well as those exposed to the external environment (i.e. skin, lung and intestines). Importantly, transcription of TLRs change as a result of an infection and the magnitude and type of TLRs expressed is based on type of invading organism (Zarember & Godowski 2002).

**Table 1:** Known human Toll‐like receptors, described agonists and potential involvement in SI‐AKI

TLR	Example of ligands	Involvement in SI‐AKI
TLR1	Triacylated lipopeptides	Pam3cys, an agonist of TLR1 complexed with TLR2 stimulates complement factor B (cfB) production in human proximal tubular cells. Experimental data suggest that this can contribute to SI‐AKI (Li *et al*. 2016)
TLR2	Bacterial: Glycolipids, phenol‐soluble modulin, lipoprotein, lipoteichoic acid peptidoglycan. Viral: proteins from measles, CMV, HSV‐1. Fungal: lipoarabinomannan, zymosan. Potential endogenous: biglycan, histones, HMBG‐1, hyaluronan, heat‐shock proteins	TLR2‐deficient mice with CLP have less renal hypoxia (Castoldi *et al*. 2012). Neutralizing histones acting via TLR2 and TLR4 reduces plasma creatinine In murine endotoxemia (Allam *et al*. 2012). Activation of TLR2 may upregulate cfB that contributes to increased mRNA level of NGAL and KIM‐1 in polymicrobial murine sepsis (Zou *et al*. 2013). TLR2 inhibits HCO^3−^ reabsorption in the medullary thick ascending limb in response to LPS (Good *et al*. 2010, 2012). Bacterial lipopeptide acts through TLR2 to increase protein permeability in cultured glomerular endothelial cells and podocytes (Pawar *et al*. 2009)
TLR3	Viral double‐stranded RNA	Activation of TLR3 may upregulate cfB that contributes to increased mRNA level of NGAL and KIM‐1 in polymicrobial murine sepsis (Zou *et al*. 2013)
TLR4	Bacterial: LPS. Fungal: Mannan. Viral: Protein from RSV. Potential endogenous: heparin sulphate, fibrinogen, biglycan, histones, HMBG‐1, hyaluronan, heat‐shock proteins	TLR4 mediates reduction in urine output and GFR in a sheep model of *E. coli* sepsis (Fenhammar *et al*. 2014). Sepsis causes TLR4 upregulation in the kidney and stimulation results in renal PMN infiltration, release of pro‐inflammatory cytokines and chemokines, glomerular endothelial swelling, tubular ion transport dysfunction and apoptosis. See text for further details and references
TLR5	Bacterial flagellin	Flagellin causes a systemic inflammatory response and liver injury but no renal injury, estimated by change in plasma urea, in mice (Liaudet *et al*. 2002). TLR5 activation may protect against urinary infection (Andersen‐Nissen *et al*. 2007)
TLR6	Interacts with TLR2 to recognize bacterial lipopeptides and fungal zymosan	Unknown
TLR7	Single‐stranded RNA from, that is HIV and Influenza virus	TLR7 has been shown to activate B‐lymphocytes and contribute to glomerulonephritis in response to viral agonists (Pawar *et al*. 2007). Although not verified to be mediated completely by TLR7, AKI often develops in patients with severe influenza infection and this is associated with increased risk of dying (Pettila *et al*. 2011).
TLR8	ssRNA, synthetic imidazoquinoline derivatives	Unknown.
TLR9	Viral and bacterial CpG DNA motifs	Knockdown of TLR9 by siRNA reduced the increase in plasma creatinine and urea in response to polymicrobial sepsis in mice (Liu *et al*. 2012). TLR9 knockout reduced AKI in CLP‐induced septic mice (Dear *et al*. 2006, Yasuda *et al*. 2008). Possibly via stimulation by mitochondrial DNA (Tsuji *et al*. 2015)
TLR10	Unknown	Unknown

TLR4 is a trans‐membrane protein with extracellular leucine‐rich repeats forming a horseshoe‐like shape (Kim *et al*. 2007). Lipopolysaccharide (LPS) is a major component of the gram‐negative bacterial cell wall and the main agonist of TLR4. On a particular cell, TLR4 is found both on the cell surface and in intracellular phagosomes where TLR4 forms a receptor complex with myeloid differentiation factor 2 (MD2) (Shimazu *et al*. 1999, Kawai & Akira 2011). Cluster of differentiation 14 (CD14), a co‐receptor, transfers LPS to the TLR4/MD2 complex as well as facilitates endocytosis of TLR4 (Zanoni *et al*. 2011, Rajaiah *et al*. 2015). LPS upregulates the production of pro‐inflammatory mediators via MyD88‐ and TRIF‐dependent pathways, which signal from the cell surface and endosomes respectively (Kagan *et al*. 2008). Both pathways cause translocation of NF‐κB to the cell nucleus resulting in production and release of cytokines and chemokines. TRIF signalling also results in activation of IRF‐3 transcribing interferons (Palsson‐McDermott & O'Neill 2004).

Human cells with TLR4 polymorphism display a reduced inflammatory response to LPS, verifying the LPS‐TLR4 connection also in humans (Tulic *et al*. 2007).

## Sepsis and AKI

More than 5% of hospitalized patients suffer from AKI and thus have an increased risk of dying or developing chronic kidney disease (Wonnacott *et al*. 2014). In intensive care, AKI is more common and affects more than 50% of the patients admitted, with the severity of renal dysfunction being strongly associated with increased hospital mortality (Hoste *et al*. 2015).

**Figure 1:**
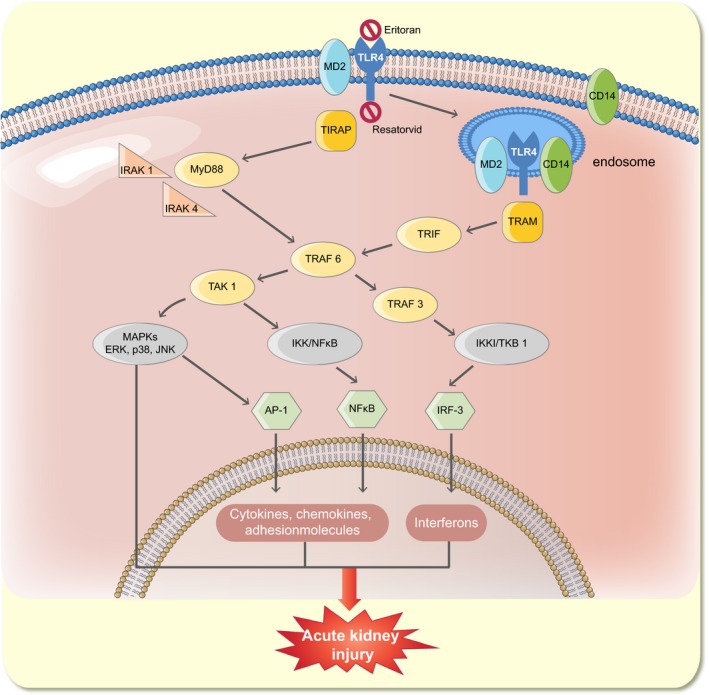
TLR4 signalling in SI‐AKI. LPS binding to the extracellular domain of TLR4 and MD2 is facilitated by CD14 and results in activation of both the MyD88‐ and TRIF‐dependent pathways. In the proximal tubule, it has been described that upon activation the entire TLR4/MD2/CD14 complex is subject to endocytosis. The downstream effects include upregulation of transcription factors NFkB, AP‐1 and IRF‐3 resulting in transcription of pro‐inflammatory genes including cytokines, chemokines, adhesion molecules and interferons. Furthermore, activation of MAPKs such as p38, ERK and JNK takes place. The ensuing overall renal effects include endothelial and tubular dysfunction in addition to altered renal metabolism and circulation (not shown in figure), giving rise to acute kidney injury. The two methods of inhibiting TLR4 signalling are shown above. Eritoran (E5564), a synthetic lipopolysaccharide, binds to cell‐surface TLR4‐MD2 without activating the receptor and thus inhibits signalling by bacterial LPS. Resatorvid (TAK‐242) binds to TLR4's intracellular domain and blocks further signalling downstream. TRIF (Toll/IL‐1 receptor domain containing adaptor inducing IFN‐β); IRF‐3 (interferon regulatory factor 3); NFkB (nuclear factor kappa‐light‐chain enhancer of activated B cells); AP‐1 (activator protein 1); TRAF (TNF receptor associated factor); IRAK (impairment of IL‐1R associated kinase 1 activity); TAK1 (transforming growth factor beta‐activated kinase 1); TBK1 (tank binding kinase 1); IKK (I‐kappa B kinase complex); MAPKs (mitogen activated protein kinases).

**Figure 2:**
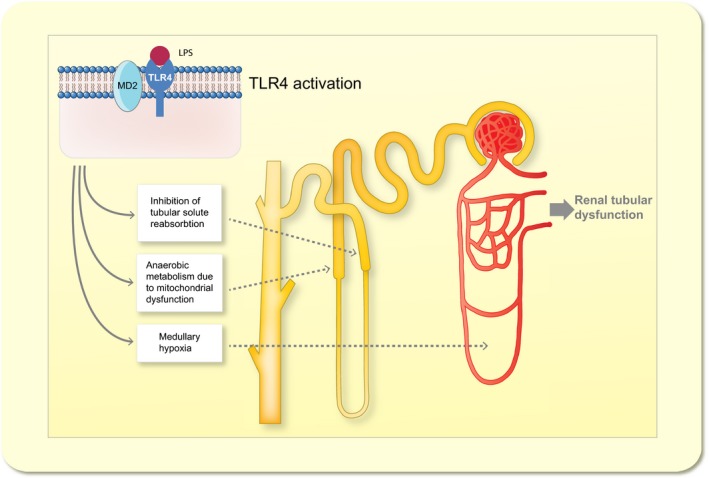
Toll‐like receptor 4 (TLR4)‐mediated renal tubular dysfunction. Selection of proposed mechanisms by which TLR4 activation contributes to tubular dysfunction in S‐AKI (for details see text). LPS (Lipopolysaccharide); TLR4 (Toll‐like receptor 4).

**Figure 3:**
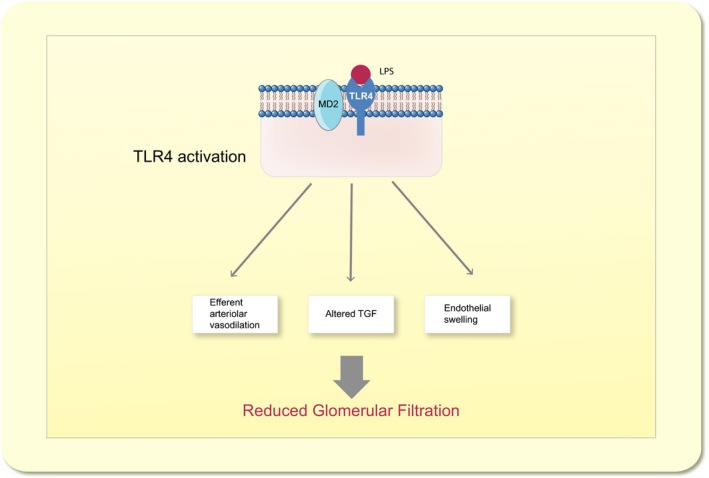
Toll‐like receptor 4 (TLR4)‐mediated reduction in glomerular filtration rate. Selection of proposed mechanisms by which TLR4 activation contributes to reduced glomerular filtration in S‐AKI (for details see text). LPS (Lipopolysaccharide); TLR4 (Toll‐like receptor 4); TGF (Tubuloglomerular feedback).

Sepsis is the most common cause of AKI in intensive care units, and in combination, sepsis and severe renal dysfunction constitute a major risk of dying (50–65% 90‐day mortality) (Bagshaw *et al*. 2007, Zang & Yan 2013, Cruz *et al*. 2014). Renal failure in sepsis is characterized by decreased GFR, often estimated by increased plasma creatinine, and oliguria/anuria (Angus & van der Poll 2013). However, the underlying mechanisms are unknown. Compared to other aetiologies of AKI, sepsis is associated with significantly higher mortality rates. This can be attributed to the fact that septic patients often suffer from various comorbidities and generally are severely ill. However, in epidemiological studies, AKI severe enough to require renal replacement therapy has been found to be an independent predictor of mortality in patients with sepsis (Sakhuja *et al*. 2015). Thus, understanding the causes of renal dysfunction in sepsis and identifying new targets for treatment is pivotal if survival is to be improved in this large group of critically ill patients (Figs 1, 2, 3).

Acute kidney injury is a functional classification and the underlying causes likely differ. Many relevant hypotheses for the mechanisms of AKI have been described (Persson 2013). The most common cause of AKI is assumed to be changes in renal perfusion causing ischaemia and acute tubular necrosis (ATN) (Hultstrom 2013). For long, this concept also included SI‐AKI (Schrier & Wang 2004). However, a significant major reduction in renal blood flow has been difficult to verify in humans with sepsis (Brenner *et al*. 1990, Prowle *et al*. 2012) and post‐mortem analysis of kidneys from patients with severe sepsis and AKI have failed to show widespread ATN (Lerolle *et al*. 2010). On the contrary, in large animal models of septic shock, it has been shown that AKI develops even though renal blood flow is increased or unchanged (Langenberg *et al*. 2006, Frithiof *et al*. 2011). This has led to the hypothesis that local renal and/or systemic inflammation contributes to SI‐AKI and that the kidney failure often seen in association with sepsis not necessarily has to involve inadequate global renal perfusion (Lipcsey & Bellomo 2011).

## Targeting TLR4 in experimental models of SI‐AKI

The recent shift in focus from SI‐AKI being a cardiovascular problem to becoming an inflammatory consequence has made TLR4 a potential mediator of SI‐AKI. Indeed, recent experimental evidence point strongly towards that TLR4 is of significant importance in the development of AKI in gram‐negative sepsis. Pre‐treatment with TAK‐242, which inhibits the effect of TLR4, in conscious sheep subjected to an intravenous LPS infusion completely abolished the AKI that developed in the vehicle treated animals (Fenhammar *et al*. 2011). In a follow‐up study Fenhammar *et al*. (2014) demonstrated that TAK‐242 reversed a pronounced decline in urine output and GFR, when used as intravenous treatment twelve hours into ovine hyperdynamic *E. coli* sepsis. This was associated with reduced endothelial swelling in the glomeruli, but compared to sham‐treated animals, there were no differences with regard to blood pressure, RBF or renal microcirculation, suggesting that hemodynamic factors were not pivotal for the SI‐AKI to develop.

Although renal function cannot be monitored with the same temporal resolution, similar detrimental effects of TLR4 as in sheep have been described in murine SI‐AKI. TLR4‐deficient mice were protected from the increase in BUN and serum creatinine caused by LPS and cisplatin in wild‐type mice (Ramesh *et al*. 2007). These results were recently confirmed in endotoxemic TLR4‐knockout mice (Smith *et al*. 2015). Furthermore, TLR4‐deficient mice subjected to polymicrobial sepsis had reduced increase in plasma creatinine and urea compared to wild‐type mice (Castoldi *et al*. 2012).

Thus, either pharmacological inhibition or genetic deletion of TLR4 significantly reduces SI‐AKI in various species and experimental models. In the following sections, we will review detailed mechanisms for how TLR4 may mediate renal dysfunction in sepsis.

## Renal expression of TLR4

Several studies have verified renal TLR4 expression in both cortex and medulla (Tsuboi *et al*. 2002, Wolfs *et al*. 2002, Zarember & Godowski 2002, Laestadius *et al*. 2003, Samuelsson *et al*. 2004, El‐Achkar *et al*. 2006, 2007, Pulskens *et al*. 2008, Zhang *et al*. 2008, Chen *et al*. 2011, Kalakeche *et al*. 2011, Liu *et al*. 2014a) On a cellular level, TLR4 is mainly located in tubular epithelium but is also found in the glomeruli, including podocytes and mesangial cells, as well as vascular endothelium (Wolfs *et al*. 2002, Brown *et al*. 2007, El‐Achkar *et al*. 2007, Chen *et al*. 2011). Renal expression appears dynamic with a low constitutive level that increases in response to LPS. El‐Achkar *et al*. could show a fluctuating expression in rodent kidneys when subjected to polymicrobial sepsis. An initially low expression was increased throughout the tubuli, glomeruli and renal vasculature followed by a slower retreat to baseline within 3 days (El‐Achkar *et al*. 2006). In general, TLR4 is often situated on the apical cell membrane and would therefore present on the luminal side suggesting optimal ligand exposure in blood, filtrate and urine respectively (Wolfs *et al*. 2002). CD14 expression has also been noted to increase in response to sepsis. Polymicrobial sepsis‐induced CD14 predominantly in proximal tubuli where it co‐localized with TLR4 at the apical cell membrane and in endosomes in rat kidneys (El‐Achkar *et al*. 2006). Internalization of TLR4 into endosomes has been shown to be mediated by both CD14‐dependent and CD14‐independent processes (Zanoni *et al*. 2011, Rajaiah *et al*. 2015).

## TLR4, cytokines and chemokines in SI‐AKI

Cytokines and chemokines are released during sepsis and implicated in the pathogenesis of SI‐AKI. Renal cells and circulating immune cells release several cytokines in response to TLR4‐LPS signalling, including pro‐inflammatory TNF‐α, IL‐6 and IL‐1β (Cunningham *et al*. 2004, Castoldi *et al*. 2012, Han *et al*. 2012). During polymicrobial sepsis, TLR4‐, TLR2‐ and MyD88‐deficient mice displayed lower levels of cytokines, in turn correlated with less renal inflammation and renal dysfunction (Castoldi *et al*. 2012). However, as mice without tubular TLR4 but with significantly increased systemic cytokine levels are protected against LPS‐induced renal injury, the local inflammatory response appears most important for SI‐AKI to occur (Hato *et al*. 2015).

Studies have shown renal chemokine release induced by TLR4 activation. Tubular epithelial cells expressed monocyte chemoattractant protein‐1 (MCP‐1) and RANTES when exposed to synthetic lipid A and released CXCL1 and CXCL2 in response to LPS (Tsuboi *et al*. 2002, Brown *et al*. 2007). Furthermore, urinary tract epithelial cells also released CXCL2 via TLR4 signalling and thus recruited leucocytes to the infected urinary tract (Patole *et al*. 2005). In addition to these inflammatory mediators, adhesion molecules are upregulated as a result of TLR4 activation. ICAM‐1 expression was increased on tubular epithelial cells and glomerular endothelial cells in murine endotoxemia (Cunningham *et al*. 2004). Hence, current data suggest that cytokines, chemokines and adhesion molecules are released due to TLR4 signalling. This contributes to renal leucocyte infiltration and renal injury in sepsis.

## Leucocyte infiltration, TLR4 and SI‐AKI

In the experimental setting, renal leucocyte infiltration is associated with renal dysfunction, and in post‐mortem biopsies, from septic patients, leucocytes were observed in the glomeruli, capillaries and tubular lumens (Lerolle *et al*. 2010). TLR4 activation has repeatedly been shown to be involved in renal inflammation, as in murine polymicrobial sepsis, murine endotoxemia and ovine *E. coli* sepsis where the phenomenon is associated with renal dysfunction (Cunningham *et al*. 2004, Castoldi *et al*. 2012, Fenhammar *et al*. 2014). Stimulated leucocytes may both participate in eradicating invading pathogens as well as causing collateral damage to renal tissues. In the previously mentioned studies, inhibiting TLR4 signalling or depleting leucocytes improved renal function. Other data support the opposite scenario where PMN depleted mice subjected to LPS displayed greater kidney injury compared to mice with normal PMN count (Wulfert *et al*. 2012).

Several groups have studied the significance of local renal or systemic TLR4 in leucocyte infiltration and renal dysfunction. In an experimental set‐up of wild‐type and TLR4‐deficient mice, single kidney transplants were made between the two strains before treatment with LPS. Wild‐type animals with TLR4‐deficient kidneys developed renal inflammation and AKI, emphasizing the need for systemic TLR4 in order for SI‐AKI to develop (Cunningham *et al*. 2004). In contrast, renal TLR4 was necessary for AKI to develop in a different murine LPS models (Hato *et al*. 2015).

In summary, activation of TLR4 promotes renal leucocyte infiltration. Data supporting both beneficial and detrimental effects on the kidney as a result of this have been described. It is reasonable to assume that an inflammatory process would be able to cause endogenous tissue damage but at the same time aid in clearing an infection that has reached the kidney.

## Renal endothelial effects of TLR4 activation

Endothelial dysfunction in sepsis is a complex event causing abnormal vascular tone, hyperpermeability, hypercoagulability and leucocyte migration (Aird 2003). Activation of TLR4 may be a part of the mechanism mediating endothelial dysfunction in the kidney. This is supported by reports demonstrating that TLR4‐deficient mice subjected to caecal ligation and puncture have an attenuated increase in renal vascular permeability (Castoldi *et al*. 2012). Furthermore, LPS‐TLR4 signalling in podocytes has been noted to cause diminished foot processes and hence proteinuria (Reiser *et al*. 2004).

Glomerular endothelial swelling and diminished glomerular endothelial fenestrae were noted in mice subjected to LPS or TNF‐α as well as in sheep subjected to live *E. coli* infusion (Fenhammar *et al*. 2014, Xu *et al*. 2015). Normal GFR is dependent on an intact and normally functioning glomerular filtration barrier, thus reduced filtration area due to these effects reduce GFR. The endothelial changes and reduction in GFR were attenuated in mice by inhibiting the effect of TNF‐α and in sheep with the TLR4 inhibitor TAK‐242.

A possible mechanism for TLR4‐induced endothelial swelling is through increased reactive oxygen species as Becerra *et al*. (2011) noted a ROS‐dependent influx of sodium causing cellular oedema in gram‐negative sepsis.

## TLR4‐induced tubular dysfunction

Tubular dysfunction is frequently described in experimental models of sepsis and is often due to tubular cell injury (Di Sole & Girardi 2011). TLR4 and LPS interactions in tubular cells cause release of inflammatory mediators (as described above), cellular uptake of LPS oxidative stress, reduction of ion reabsorption and reduced tubular flow.

Systemically administered LPS in rats reaches the kidneys within minutes and although homogenously filtered, LPS distributes heterogeneously amongst the nephrons (El‐Achkar *et al*. 2006). Furthermore, LPS interacts differently with distinct tubular sections. In S1 tubular segments, systemically administered LPS is selectively taken up and neutralized after filtration into the lumen via TLR4‐CD14 influenced endocytosis, yet injures tubular segments downstream through TLR4‐dependent oxidative stress (Kalakeche *et al*. 2011, Zanoni *et al*. 2011).

It has also been suggested that the tubular cells adapt to cellular stress in sepsis by conserving energy and thus prioritizing cell survival above maintaining organ function (Gomez *et al*. 2014). Sodium transport is the major energy‐consuming process in tubular epithelial cells; thus, reducing sodium transport activity decreases energy expenditure and promotes cell survival (Gomez *et al*. 2015). Downregulation of renal sodium, chloride and glucose transporters in response to LPS and inflammatory cytokines has been described (Schmidt *et al*. 2007a,b,c). Later reports have demonstrated TLR4 as directly involved in inhibition of bicarbonate absorption in the medullary thick ascending limb (mTAL) (Watts *et al*. 2011, 2013b, Good *et al*. 2012). A reduction in chloride and sodium reabsorption during sepsis would in turn reduce GFR through tubuloglomerular feedback (Morrell *et al*. 2014). In a recent study in endotoxemic mice, reduced tubular flow was demonstrated to be TLR4‐dependent. LPS accumulation in proximal tubular lumens was seen within hours of administration, and within 1 day, luminal obstruction by LPS was observed. This mechanism may contribute to reduced urine output in gram‐negative sepsis (Nakano *et al*. 2015). To summarize, LPS‐induced TLR4 activation in tubular cells seems to entail inflammatory, adaptive as well as directly injurious effects.

## TLR4, renal apoptosis and acute tubular necrosis

TLR4 activation may cause tubular cell injury, as mentioned above, yet the available human material to date displays renal histopathological changes in SI‐AKI discordant with the often pronounced loss of renal function (Takasu *et al*. 2013). Acute tubular necrosis (ATN) is commonly seen in ischaemic kidney injury but not in SI‐AKI. In a systemic review, ATN was only noted in 25 of 184 patient samples with SI‐AKI (Langenberg *et al*. 2008). Two later studies with post‐mortem biopsies from patients with SI‐AKI have both observed moderate tubular injury in a majority of patients. Lerolle *et al*. (2010) noted loss of brush border, flattening of cytoplasm, tubular vacuolization and mitochondrial injury. Takasu *et al*. noted merely focal tubular injury with two distinct histological profiles correlated with the expression of kidney injury molecule‐1 (KIM‐1) and mammalian target of rapamycin (mTOR) respectively (Lerolle *et al*. 2010, Takasu *et al*. 2013). Expression of both mTOR and KIM‐1 has been described as a downstream effect of TLR4 activation (Castoldi *et al*. 2012, Watts *et al*. 2013a). Thus, tubular necrosis is rare and tubular injury is observed although not extensive enough to explain the renal dysfunction seen in SI‐AKI.

The role of apoptosis in SI‐AKI is debated. Lerolle *et al*. found increased apoptosis in the tubuli but Takasu *et al*. could not (Lerolle *et al*. 2010, Takasu *et al*. 2013). Data from animal models are also inconsistent. Several experimental settings have found increased apoptosis in tubular epithelial cells, including murine endotoxemia and murine polymicrobial sepsis (Cunningham *et al*. 2004, Castoldi *et al*. 2012, Lee *et al*. 2012). Further, pro‐apoptotic signalling by TLR4 has been proposed including caspase induction (Cunningham *et al*. 2004, Castoldi *et al*. 2012, Liu *et al*. 2014b, Hsu *et al*. 2016), However, other studies involving ovine *E. coli* sepsis and murine polymicrobial sepsis could not show increased apoptosis (Dear *et al*. 2006, Langenberg *et al*. 2014). In human SI‐AKI, neither necrosis nor apoptosis are a dominant feature making the significance of the experimental observations of those events uncertain. The limited histological damage seen in human SI‐AKI may perhaps be explained by a TLR4‐mediated adaptive mechanism as discussed previously. SI‐AKI could also be mainly a physiological and not a morphological problem.

## Renal macrocirculatory alterations as cause of SI‐AKI

Sepsis‐induced acute kidney injury (SI‐AKI) has traditionally been viewed as a result of hypoperfusion due to distributional hypovolemia and shock (Schrier & Wang 2004). Experimental and clinical observations have challenged this view, and a more complex perspective including cellular dysfunction and microcirculatory alterations has recently been postulated (Gomez *et al*. 2014). Mediators such as nitric oxide (NO), cytokines and reactive oxygen species (ROS) induce alterations in macro‐ and microcirculation and are released due to TLR4 activation (Patzak *et al*. 2004, Holmqvist *et al*. 2005).

Clinical observational studies have detected a correlation between lower mean arterial pressure (MAP) and the risk of progression of AKI (Poukkanen *et al*. 2013). There is also a correlation between the aggregated time of hypotension defined as MAP < 65 and the risk of progression of AKI (Janssen van Doorn *et al*. 2014). In experimental studies, shorter periods of hypoperfusion alone seldom result in AKI (Saotome *et al*. 2010). In the clinical setting, the observation of a low frequency of AKI after cardiac arrests supports this view (Chua *et al*. 2012). The susceptibility to low arterial blood pressure might, however, differ in an inflamed kidney where hypotension might be considered a second insult. As hypotension is a common feature of septic shock, it might be a clinically relevant mechanism of SI‐AKI development. In a large randomized controlled study of different target blood pressures (MAP 65–70 vs. 80–85), a decreased risk of AKI with higher blood pressures was observed in patients with a previous history of hypertension but not otherwise in healthy adults (Asfar *et al*. 2014). Fenhammar *et al*. (2011) reported that TLR4 inhibition both prevented LPS‐induced hypotension and AKI but merely treating the low blood pressure with vasopressors had no effect on the development of renal dysfunction. This indicates that hypotension is not the sole cause of TLR4‐mediated SI‐AKI. Data further supporting this view was presented in a recent porcine experimental sepsis study were renal failure developed in some animals but not others, even though perfusion pressure was identical in both groups (Benes *et al*. 2011).

Large animal experiments have described an early phase of septic AKI with normal or increased renal blood flow (RBF) during sepsis where renal hypoperfusion in a global sense reasonably cannot explain the reduction in GFR (Langenberg *et al*. 2006). Haemodynamically, this hyperdynamic state is characterized by an increased cardiac output (CO) and a low systemic vascular resistance, similar to what is commonly observed in early clinical sepsis (Langenberg *et al*. 2005). Human data of RBF during sepsis are scarce as the unreliability of PAH clearance in sepsis until recently mandated for more invasive measuring (Brenner *et al*. 1990, Prowle *et al*. 2012). In the studies by Brenner *et al*. and Prowle *et al*., including a total of 18 septic patients, absolute values of RBF exhibit large variations, although some common patterns were observed. The renal fraction of the cardiac output was decreased and was by Brenner *et al*. suggested to correlate to GFR, although the small number of participating patients makes this conclusion uncertain. Whereas high CO and AKI in early human sepsis seem to be common, animal models of septic AKI can be categorized as either hyper‐ or hypodynamic depending on the effects of CO (Langenberg *et al*. 2005). Hypodynamic circulation is most often accompanied by reduced RBF where hyperdynamic models often exhibit less affected or increased RBF. In the previous mentioned ovine study, where sepsis was induced by *E. coli* infusion, TLR4 inhibition significantly improved renal function without affecting renal blood flow (Fenhammar *et al*. 2014).

## Renocirculatory dysregulation in S‐AKI

There are several proposed mechanisms that might further explain the macrocirculatory alterations and their relevance to renal function.

Glomerular filtration is dependent of the perfusion pressure regulated by the tone of the afferent and efferent arteriole. A reduced renal blood flow, either caused by low systemic pressure or increased intrarenal resistance due to pathological afferent arteriolar constriction, may reduce GFR due to lowered filtration pressure. In the hyperdynamic models with increased RBF, filtration pressure has not been directly assessed to our knowledge, but may theoretically be reduced as well. The efferent arterial tone is crucial for maintaining an adequate filtration pressure, and several mediators linked to TLR4 activation such as NO, prostaglandins and endothelin are known to influence vascular tone (El‐Achkar *et al*. 2007, Fenhammar *et al*. 2008). During septic shock, there is a massive release of NO. NO counteracts the constricting effects of angiotensin II on the efferent arteriole (Patzak *et al*. 2004). This may cause a reduction in the efferent arteriolar tone thus reducing filtration pressure resulting in the typical decrease in urine output and increase in plasma creatinine observed in SI‐AKI. Improvement of renal function after angiotensin II administration, although RBF is decreased, further strengthens this hypothesis (Wan *et al*. 2009). Increased expression of iNOS and eNOS in these septic models is found in the renal cortex with a paradoxically decreased expression in the renal medulla (Langenberg *et al*. 2014). This contrasting difference in expression pattern may explain why blockade of iNOS do not restore renal function (Ishikawa *et al*. 2012).

Increased intrarenal vascular resistance is, however, also linked to septic AKI in man (Dewitte *et al*. 2012). Some endotoxemic large animal models (Fenhammar *et al*. 2008) and small animal models (Nitescu *et al*. 2008) improve renal function by mainly vasodilatory interventions, indicating renal vasoconstriction by TLR4. This may be due to a hypodynamic experimental phenotype, but an increased intrarenal resistance is also demonstrated in hyperdynamic septic pigs with AKI, compared with those lacking AKI (Benes *et al*. 2011). An increase in intrarenal resistance has been suggested to reflect a different mechanism in septic AKI which may be more dominant later in the clinical course because manifest AKI in man usually is accompanied by a reduced RBF (Prowle *et al*. 2012).

One of the key features of the renal circulation is autoregulation of blood flow within a wide range of systemic blood pressures. Clinical data suggest that this intrinsic control may be disrupted during sepsis where renal blood flow seems to correlate to cardiac output (Prowle *et al*. 2012). It has been reported that endotoxemia attenuates the dynamic response of the tubuloglomerular feedback mechanism (TGF) in rats (Nitescu *et al*. 2010). TLR4 might be a key component as CLP‐treated mice upregulates cTAL COX‐2 and while developing AKI, but not if TLR4 deficient (El‐Achkar *et al*. 2007). COX‐2 upregulation in cTAL and adjacent macula densa affects the TGF in a proconstrictive manner (Araujo & Welch 2009).

## Altered microcirculation during endotoxemia and TLR4 activation

As mentioned earlier, renal tubular cells express TLR4 and can be directly activated by LPS. Also, an impaired microcirculation in the peri‐tubular capillary network has been described during endotoxemia in mice (Tiwari *et al*. 2005, Wu *et al*. 2007). Others report a peri‐tubular hypervelocity of red blood cells (Burban *et al*. 2013) during CLP‐induced sepsis in rat with a reduction in GFR, which in turn was reversed with noradrenaline infusion. Peri‐tubular capillary dysfunction is suggested to be an early feature of SI‐AKI in mice after CLP (Wang *et al*. 2012). Reduced tubular flow and impaired peri‐tubular microcirculation resulting in AKI have recently been demonstrated in LPS‐treated mice and are likely TLR4 dependent, but apparently by a pathway independent of TNF‐α (Nakano *et al*. 2015).

Several authors have investigated changes in renal microcirculation in general and if this is linked to the development of SI‐AKI. There are a number of methods to assess microcirculation, which may explain the variability in the findings. Authors using laser Doppler probes (Di Giantomasso *et al*. 2003, Porta *et al*. 2006, Chvojka *et al*. 2008, Fenhammar *et al*. 2014, Calzavacca *et al*. 2015) have described both variable, regionally reduced and regionally increased tissue perfusion in their experiments. Interestingly, TLR4 antagonism did not increase neither cortical nor medullar perfusion while still attenuating AKI (Fenhammar *et al*. 2014). Kidneys of endotoxemic rats evaluated with laser speckle imaging after fluid resuscitation present a pattern of increased heterogeneity regarding microcirculation (Legrand *et al*. 2011). There are both hypoperfused and normally perfused areas in conjunction with leucocyte infiltration and increased iNOS expression. These findings have generated a hypothesis of a heterogenous microcirculatory defect in gram‐negative SI‐AKI, where hypoperfused areas generate micro‐ischaemia despite normal total renal blood flow (Gomez *et al*. 2014). This may in turn explain differences and variability measured with laser Doppler probes besides different experimental design.

## Renal oxygenation after TLR4 activation

In human postoperative AKI, there is a characteristic change in oxygen demand in comparison with sodium reabsorption (Redfors *et al*. 2010). Sodium resorption is the result of the most oxygen demanding process in the kidney and oxygen demand per reabsorbed molecule of sodium increases during AKI in a typical manner. Similar findings are observed in fluid resuscitated endotoxemic rats (Johannes *et al*. 2006) and may be commonplace in AKI in general. During the initial hours after LPS administration, oxygen consumption, QO_2_ or VO_2_, seems to remain fairly unchanged in both small and large animals (Johannes *et al*. 2006, Porta *et al*. 2006, Dyson *et al*. 2011). An exception is non‐resuscitated animal models of sepsis, both large and small, where significant reduction in RBF and oxygen delivery (DO_2_) occurs (Gullichsen *et al*. 1989, Johannes *et al*. 2009a). Increasing arterio‐venous oxygen difference has been described in the initial phase of endotoxemia (Gullichsen *et al*. 1989) and may to a certain extent compensate a reduced oxygen delivery by increasing oxygen extraction (Porta *et al*. 2006). Whereas renal QO_2_ may be measured quite reliably, the actual demand from a cellular or regional perspective is not always identical. Microscopic hypoxic areas have been described (Johannes *et al*. 2009a,b, Dyson *et al*. 2011), at least in conjuncture with reduced RBF, and this has been proposed to contribute to the development of AKI. Intrarenal oxygen shunting (Leong *et al*. 2007) is suggested to maintain tissue oxygenation constant during normal physiologic conditions and preventing hyperoxia despite altered renal blood flows necessary for GFR regulation. Besides overall renal DO_2_ and QO_2_, this would be a third determinant of tissue oxygenation. BOLD‐MRI is used to determine oxygenation in tissue, but intrarenal oxygen shunting may add uncertainty to this measurement in the kidney (Evans *et al*. 2007, Niendorf *et al*. 2015). BOLD‐MRI was used to demonstrate intact tissue oxygenation after 18 h LPS and TLR4 stimulation in mice (Tran *et al*. 2011), but in that study the lack of hypoxia was also indicated by an absence of elevated HIF‐1‐α expression, which is a significant marker for tubular hypoxia. However, regionally lower tissue oxygenation in the medulla paired with lower medullary perfusion is found in hyperdynamic gram‐negative sepsis in sheep (Calzavacca *et al*. 2015). This despite elevated RBF and maintained cortical oxygenation and perfusion, which may reflect intrarenal redistribution of blood flow during sepsis.

## TLR4 activation and renal oxygen utilization

Lactate/pyruvate ratios reflect anaerobic metabolism and when increased imply a difference between actual demand and consumption of oxygen. Increased intrarenal ratios have been observed both cortically and medullary after TLR4 activation by either *E. coli* or LPS (Levy *et al*. 2003, Fenhammar *et al*. 2014). TLR4 blocking during *E. coli* infusion attenuates the increased lactate/pyruvate ratio and restores renal function. In LPS‐treated rats (Levy *et al*. 2003), the increased lactate/pyruvate ratio reflects an actual decrease in intrarenal ATP levels when accompanied by a profound reduction in RBF.

Increased anaerobic metabolism could theoretically occur despite normal oxygen delivery if mitochondrial function is impaired. As the kidney is second only to the heart in mitochondrial density, a role for mitochondrial dysfunction in septic AKI seems plausible. However, when Porta *et al*. (2006) investigated mitochondrial dysfunction in pigs after 24 h of TLR4 stimulation by LPS infusion, no mitochondrial dysfunction was found in the kidney. Others have described morphological changes of the proximal tubular mitochondria after LPS exposure in mice, such as swelling and rarefied cristae, and these have been linked to functional impairment assessed by cytochrome c oxidase activity, the last enzyme in the respiratory electron transport chain (Tran *et al*. 2011). The same authors described that mice lacking PGC‐1‐α, an important regulator of, mitochondrial biogenesis, exhibited a lack of recovery in renal function after TLR4 activation by LPS administration. Interestingly, mice exposed to LPS developed AKI where LPS seemed to suppress PGC‐1‐α and contribute to mitochondrial dysfunction (Smith *et al*. 2015). This was not observed in TLR4‐deficient mice who also maintained normal renal function. Autophagy is also induced in renal tubular epithelial cells by LPS through a TLR4‐dependent mechanism and seems to contribute to renoprotection (Leventhal *et al*. 2016). Renoprotective effects by autophagy have also been supported in other models of SI‐AKI (Hsiao *et al*. 2012). Species and experimental phenotype may explain different findings regarding mitochondrial dysfunction. In the experiments by Tran *et al.,* RBF decreased early and significantly, in contrast to the study by Porta *et al.,* which may explain the different findings. Also, in the later, GFR is not assessed and may therefore reflect a lack of AKI development.

## Clinical trials of substances targeting TLR4 in sepsis

Two compounds interfering with LPS‐TLR4 signalling have been used in clinical trials: Eritoran (also known as E5564) and Resatorvid (also known as TAK‐242); however, none have focused specifically on SI‐AKI. Eritoran is a synthetically produced lipopolysaccharide that binds to cell‐surface TLR4‐MD2 receptor without activating it and thereby blocks the effects of bacterial LPS. Resatorvid attaches to the intracellular domain of TLR4 and inhibits the signal transduction leading to NFκB activation. A randomized, double‐blind, placebo‐controlled trial of TAK‐242 showed a trend towards a reduced 28‐day mortality in patients with both septic shock and respiratory failure in the treatment group, but the result was not significant (Rice *et al*. 2010). No data on renal function were presented separately, but the Sequential Organ Failure Assessment score (SOFA, in which plasma creatinine is included) did not differ between groups. Eritoran was first investigated in a prospective, randomized, double‐blind, placebo‐controlled, multi‐centre, ascending‐dose trial in which a high dose (105 mg) showed a tendency to reduce mortality in patients with severe sepsis (Tidswell *et al*. 2010). In a follow‐up phase III study, Eritoran did not increase survival in septic patients compared to placebo (Opal *et al*. 2013). Furthermore, patients with SI‐AKI did not have reduced mortality if treated with Eritoran. This may be due to the facts that also gram‐positive infections were treated with Eritoran, a relatively low mortality rate in both the treatment and placebo group, treatment was initiated too late or that low levels of circulating LPS was present. It may also indicate that TLR4 activation does not cause AKI in these patients or that separate inflammatory mediators, acting via pathways different from TLR4, also contributes to SI‐AKI. Different study designs may, however, truly test the hypothesis that inhibition of TLR4 attenuates SI‐AKI in human gram‐negative sepsis.

## Conclusion

TLR4 is central in the inflammatory signalling cascade triggered by infection, as it constitutes the main sensor of gram‐negative bacteria, which initiates an immune reaction causing host damage. In the experimental setting, TLR4 stimulation of the innate immune system can cause renal injury and dysfunction. What is more, TLR4‐signalling blockade in various sepsis models blunts or even abolishes AKI.

Experimentally, TLR4 activation entails both glomerular and tubular effects reducing GFR and impairing tubular function. Glomerular endothelial swelling in combination with decreased filtration pressure (due to either pre‐glomerular vasoconstriction or post‐glomerular vasodilation) plays a role in diminishing GFR. TLR4‐mediated mitochondrial dysfunction and an adaptive reduction in bicarbonate reabsorption further compromises tubular function. In human sepsis, the mechanisms underlying renal dysfunction remain unknown, and so is the exact role of LPS mediated TLR4‐activation. For completely blocking the detrimental effects of the immune system in SI‐AKI, it appears likely that further signalling pathways other than those elicited via TLR4 require targeting.

Today, plenty pre‐clinical data support targeting TLR4 for preventing or treating AKI in human gram‐negative sepsis. Strict selection of patients with gram‐negative infections and assessment of circulating LPS is recommended at this stage.

## Conflict of interest

The authors report no conflicting interests.

We would like to thank the Stiftelsen Nordisk Fysiologi (SNF) and the German Research Foundation (Deutsche Forschungsgemeinschaft, DFG) for their generous support for the AP Symposium on ‘RENOPROTECTION’. RF was supported by funds from the Swedish Research Council (grant 523‐2014‐2569).
